# Screening for diabetes mellitus type 1 in the pediatric general population: an ethical analysis

**DOI:** 10.1007/s00431-026-06832-7

**Published:** 2026-03-13

**Authors:** P. Lechsner, R. W. Holl, F. Steger

**Affiliations:** 1 Institute of the History, Philosophy and Ethics of Medicine, German Center for Child and Adolescent Health (DZKJ), Ulm University, Partner Site Ulm, Ulm, Germany; 2 Institute of Epidemiology and Medical Biometry, German Center for Child and Adolescent Health (DZKJ), Ulm University, Partner Site Ulm, Ulm, Germany

**Keywords:** Diabetes mellitus, type 1, Autoantibodies, Mass screening, Ethics

## Abstract

Autoantibodies can predict clinical type 1 diabetes, impacting early diagnosis, prevention, and management. While integrating antibody testing into routine practice is debated globally, ethical considerations are often overlooked. This paper explores these ethical concerns through qualitative analysis based on a literature review. A PubMed literature search provided the foundation for a qualitative ethical analysis. Arguments regarding type 1 diabetes screening were extracted from 92 eligible articles and categorized using Beauchamp and Childress’s four ethical principles. A thematic and principle-oriented ethical analysis was conducted. Key ethical concerns include decision-making for children, risk determination, screening timepoints, psychological aspects, DKA rates, treatment options, and monetary and personnel aspects. Addressing these issues ensures general population screening aligns with autonomy, beneficence, nonmaleficence, and justice.

*Conclusions;* General population antibody screening for type 1 diabetes presents ethical challenges requiring careful consideration. While early detection offers benefits, risks such as psychological distress, stigmatization, and resource diversion must be taken into consideration. Further research should assess feasibility, including human resource demands, parental and child anxiety, optimal screening timepoints, development of educational material, children’s involvement in screening, and expanding options for prevention, including teplizumab approval in the EU.

**Table:** 

**What is Known:**	
• *Autoantibodies can identify individuals at risk for Type 1 Diabetes before symptom onset, and population screening is increasingly discussed as a strategy to reduce complications such as diabetic ketoacidosis at diagnosis.*	
**What is New:**	
• *This study provides a structured ethical analysis of general population antibody screening for Type 1 Diabetes using the four-principle framework of Beauchamp and Childress (autonomy, beneficence, nonmaleficence, justice)*	
• *It identifies key ethical domains, including decision-making for children, psychological impact, screening timing, resource allocation, and emerging preventive options such as Teplizumab, that should guide future implementation and research on population screening programs*

## Introduction

Type 1 diabetes (T1DM) is a chronic autoimmune disorder characterized by the destruction of insulin-producing beta cells in the pancreas and is often diagnosed in children and young adults. Worldwide, an estimated 9.2 million people are diagnosed with T1DM [1]. In Germany, around 375,000 patients have T1DM and around 4100 children develop T1DM per year [2]. In addition to serious long-term complications, T1DM can lead to life-threatening diabetic ketoacidosis (DKA), making early diagnosis and treatment crucial. Nearly 30% of children present with DKA at onset of T1DM, with significant variation across countries and generally increasing incidence over time [3]. Five main autoantibodies have been identified that can predict the onset of T1DM: islet cell antibodies (ICA), insulin autoantibodies (IAA), glutamic acid decarboxylase antibodies (GADA), thyrosinphosphatase 2 (IA2) antibodies, and zinc transporter 8 autoantibodies (ZnT8A) [4]. The ability to detect and quantitate these antibodies has significant implications for early diagnosis, preventive strategies, and clinical management. Recently, the integration of antibody testing into routine clinical practice to detect asymptomatic stages of T1DM has been discussed in several countries, including Germany. Italy is currently implementing a general population screening [5], and a multitude of countries have programs in development, including the ADIR study in Israel, the ASK study in Colorado (USA), and the Fr1da study in Germany [6]. Most discussions overlook ethical implications, which deserve thorough examination. Although earlier publications have examined the ethical implications of predictive screening for T1DM in newborns and children (e.g., Ross [7]), substantial technological advances have since occurred, such as improved antibody assays, the ability to reliably detect ZnT8, the possibility to use capillary blood for screening purposes, decreased screening costs and high-throughput screening abilities, and improved reproducibility with less false-positive results [4, 8–10]. These developments have increased the feasibility and also accuracy of screening and shifted the discussion beyond the research setting towards potential general population screening. This paper aims to elucidate ethical considerations based on a comprehensive literature search.

## Materials and methods

**Fig. 1:**
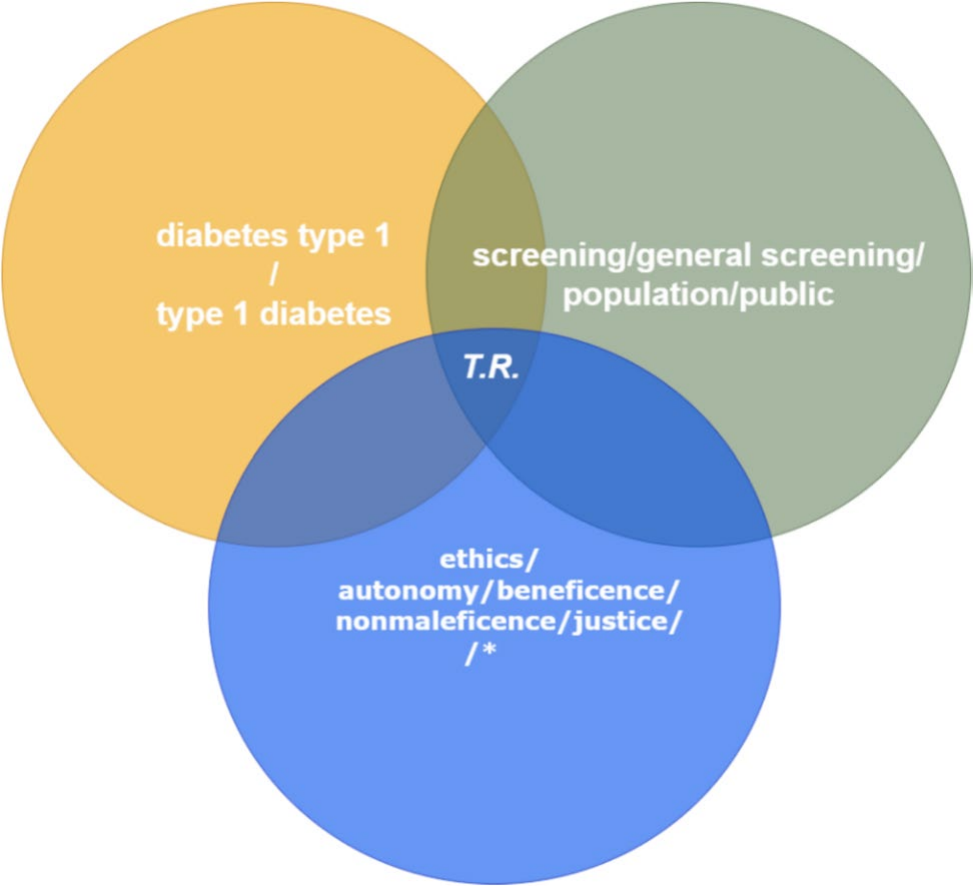
Venn diagram showing the target research (T.R.) articles of the literature research that included keywords from all three groups (the asterisk denotes that only the main keywords from this category are shown; the full list is found in the text)

**Fig. 2:**
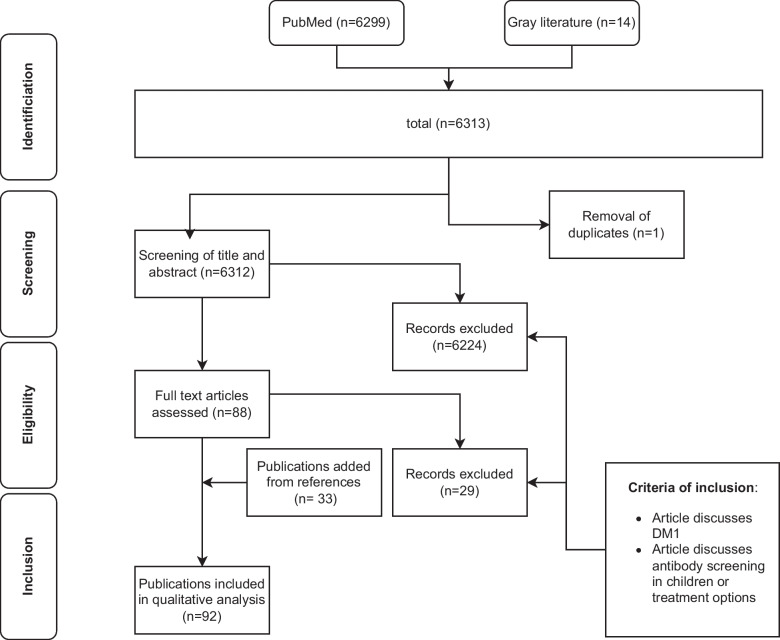
Flowchart representation of each phase of the literature research with the corresponding number of articles included/excluded

We performed a literature search with a qualitative ethical analysis. PubMed and grey literature were searched using the keywords “screening” or “general screening” or “public” or “population” (group 1) with “diabetes type 1” or “type 1 diabetes” (group 2) and with the keywords “ethics” or “ethical” or “moral” or “patient information” or “informed consent” or “autonomy” or “benefits” or “risks” or “justice” or “equity” or “fairness” or “transparency” or “data privacy” or “patient empowerment” or “responsibility” or “bias” or “beneficence” or “nonmaleficence” (group 3). For an article to be included in the first step (identification), at least one keyword from each group was required; groups 1 and 2 were used to set the general theme and group 3 to narrow down the results to relevant articles. Figure 1 presents a Venn diagram illustrating these keywords and their grouping. As T1DM antibody screening on the population level has only recently emerged, we limited the dates of publication to 2018–2024 to improve the relevance of results. After initial identification, duplicates were removed, and articles were manually screened using title and abstract. Inclusion criteria were the explicit reference to T1DM and its antibody screening in children or treatment of presymptomatic stages of T1DM. Eligible articles were assessed in their entirety. Additional articles, identified through the reference lists of publications retrieved, were included as well if they met the inclusion criteria, even if they were outside the date range. After that, all general population T1DM screening arguments were extracted and assigned to the four biomedical principles of Beauchamp and Childress (beneficence, nonmaleficence, justice, autonomy) by the main author; in cases where the extraction or assignment was not straightforward, the classification was deliberated collaboratively by the full research team to ensure consensus and methodological rigor. From a total of 6299 articles, 59 remained, plus another 33 articles identified during the assessment of the full-text articles. In total, 92 publications were included in the qualitative analysis. Figure 2 provides a flowchart representation of the process. The qualitative analysis comprised a thematic analysis, which is a qualitative method to identify, analyze, and report common patterns [11, 12]. The focus was on patterns and topics that related to the four ethical principles described above. Subsequently, the topics were ethically analyzed using Beauchamp and Childress’ principle-oriented approach [13], assessing the consistency or conflict with the bioethical categories. Finally, the findings were organized, structured, and synthesized.

## Results

**Fig. 3:**
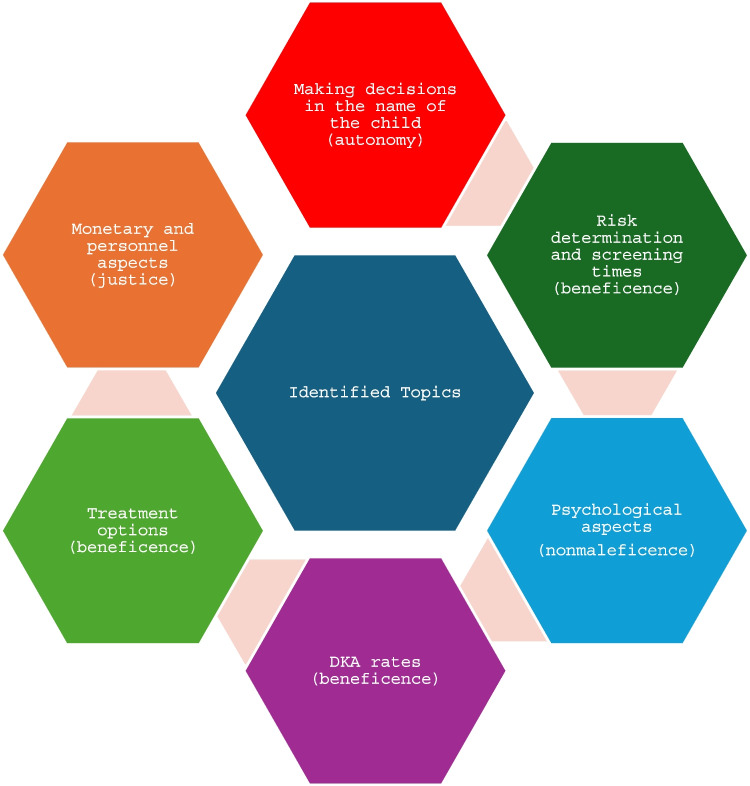
Graphical representation of the key themes identified during the literature research; shown with their associated ethical principle

As shown in Fig. 3, the main topics of ethical interest are decision-making in the name of the child, risk determination, screening scheme (age at screening and number of screenings), psychological aspects, DKA rates, preventive treatment options, and financial and personnel resources. In approximately chronological order, Fig. 3 shows how these topics would affect child and society, as well as the ethical principles that apply.

### Making decisions in the name of the child

One of the primary challenges concerning antibody screening lies in the fact that the patients involved are children who cannot make their own decisions regarding participation in the screening [14]. Instead, this decision lies with the legal guardian, usually the parents, but alternative custodial arrangements may apply. Children, like adults, have the right not to know their medical information. In cases where their welfare is at risk, the state may have to intervene and, if necessary, mandate diagnostic assessment and therapy. At the young age when screening is conducted (usually under 6 years old), the decision to participate is made by the parents/legal guardians. Generally, if they consent to the antibody screening, the resulting knowledge becomes irreversible, leaving the child to live with that awareness [14]. This is assuming that the child is informed of the test results, which might not always be the case. Either way, they will be raised in an environment in which the result is known. This can interfere with the child’s quality of life, through stigma due to “(over)-medicalization,” health anxiety and changes in parents’ behavior. Additionally, it can jeopardize the child’s “innocence of childhood” and their right to an “open future” [15]. Currently, population screening is considered at the ages of 2 and 6, since multiple cohort studies have shown screening at these ages to be sensitive and efficient, providing a practical foundation for public health implementation. However, country-specific adjustments may be necessary, taking into account population-specific disease characteristics [16, 17]. Screening at these ages makes active involvement of the children in the decision-making process basically impossible.

### Risk determination and screening scheme

Children with multiple positive autoantibodies have a 75% chance to develop clinical T1DM within 10 years, and if they develop dysglycemia (impaired fasting glucose, impaired glucose tolerance = stage 2), the 2-year risk approaches 100% [17–19]. Individual progression risk depends on multiple factors, including antibody type and number. For example, children who test positive for a single autoantibody are less likely (10–15%) to develop overt T1DM (stage 3) within 10 years [20]. Risk stratification, including age, gender, metabolic status such as insulin, C-peptide, and glucose levels, HLA genotype/polygenic risk score, BMI and other characteristics, and risk calculators or AI models can help to improve predictions [21–24]. Not every patient with multiple autoantibodies progresses to clinical T1DM, especially children without dysglycemia (stage 1), highlighting the need for informed screening [24]. Regression of multiple autoantibodies is rare (< 1% according to TEDDY study), and the time interval until stage 3 varies from months to decades [14, 25, 26]. Optimal screening schedules are still debated, particularly regarding age at initial screening and frequency of rescreening, with recommendations ranging from annually to every 3 years for high-risk children with familial disposition [27]. T1DI (Type 1 Diabetes Intelligence) and TEDDY suggest screening at age 4, which identifies 40% of cases that develop diabetes by age 15, while screening at ages 2 and 6 increases sensitivity to 82% and positive predictive value to 79%. Specificity in this study ranged from 39 to 59% and negative predictive value from 96 to 100%, depending on whether a single screening or two screenings at different ages were performed [17, 28]. Screening at age 2 would still miss cases with earlier onset, when DKA risk is highest [29], failing to “protect” children below the age of 2 from DKA regardless of whether screening is being done. Negative antibody screenings may also create false reassurances, delaying symptom recognition [14].

### Psychological aspects

Parental and child anxiety is a key concern in T1DM antibody screening. The Fr1da study reported that while parents of antibody-positive children initially showed elevated PHQ-9 depression scores, anxiety returned to baseline after ~ 6 months. In exchange for short-term stress, the family gains time to prepare for the possible challenges of T1DM treatment [14]. The Fr1da and DiMelli studies suggest that the diagnosis of early stages (stage 1 or 2) causes less distress than a clinical T1DM diagnosis [30], while the ASK study in Colorado reports significant psychological distress. For the DiPiS study, the participation of the child itself did not raise anxiety levels in the parents, but positive screening results did. Also, a lower parental education level was associated with more anxiety, while other sociodemographic factors did not appear to have an impact [31]. It is important to note that T1DM screening merely identifies the risk status of the child, but no secondary tests are available to confirm the disease early. Merely knowing about the risk status without the disease present and uncertainty about disease onset may also be at root for greater anxiety levels. Parental anxiety associated with a positive screening test of their child might also depend on how the results are communicated. In the DiPiS study, this was done by a nurse via a telephone call, unless the test was positive for at least two antibodies, in which case a pediatrician communicated the results in person. Parents perceiving their child to be at high risk remained more anxious even after 5 years of participating in the DiPiS study [31]. However, anxiety levels vary, mainly depending on the screening outcome, with parents of children testing positive for multiple autoantibodies experiencing the highest stress [32]. A positive screening without metabolic symptoms may take away a child’s “carefree” years, as not all cases progress within 10 years [14, 19]. There is a concept by Buchbinder and Timmermans called “patients in waiting,” while originally discussed for genomic screening, applies here as well. The patient “hover[s] for extended periods of time under medical attention between sickness and health” [33]. Besides regular metabolic monitoring and frequent doctor visits, the unknown latency period can adversely impact the child’s quality of life and psychological development [14]. Additionally, parents may attempt inappropriate interventions, such as unsuitable dietary restrictions, medical interventions, or overprotective behaviors [14, 34]. When asked, parents expressed significant concern about anxiety, negative behavior changes, and decreased quality of life following a positive screening test [34]. Many parents felt the burden of knowing their child would develop a lifelong condition, potentially affecting life choices, career decisions, and insurance policies [15]. It was proposed that the anxiety is heightened by the lack of treatment options and uncertainty, as unpredictable stressors are among the most challenging for parents of medically ill children [32]. Parental stress in children already diagnosed with T1DM has been more extensively studied and is linked to poorer HbA1c control and increased depressive symptoms in children [35, 36]. While this link between negative symptoms in children and parental stress might not necessarily be true for the general population, this warrants further investigation, as no identified studies examined anxiety or stress in the screened children themselves; so far, the focus has been on the parents.

### DKA rates

Children in the Fr1da study showed a DKA prevalence of < 5% compared to 20–30% in the general population in Germany and 40–60% in the USA [18]. While DKA rates are lower in familial T1DM patients, probably due to higher disease awareness, it is likely that all pre-diabetic patients would benefit from close follow-up regardless of family history [37, 38]. Preventing DKA in children not only shows acute benefits but also better long-term glycemic control [39, 40], reducing HbA1c by ~ 1.4%, persisting for up to 15 years after diagnosis [40]. Similarly, the Swedish Paediatric Diabetes Quality Registry (SWEDIABKIDS) and the Swedish National Diabetes Registry (NDR) showed that better metabolic control at diagnosis predicted better metabolic control later in life [41]. An unaddressed limitation concerns individuals with a single positive islet autoantibody. Despite a substantially increased risk of progression to T1DM, the Fr1da study does not report this test result. Failure to adequately communicate residual risk may engender a false sense of security, potentially delaying symptom recognition and increasing the risk of DKA at clinical presentation. Additionally, B. Karges states that DKA reduction is not necessarily achieved through antibody testing itself but through educating parents and children [26]. Furthermore, it remains uncertain whether this reduction in DKA would persist in the general population as existing studies mainly involved highly motivated participants with greater health awareness.

### Treatment options

Several studies highlight teplizumab, a monoclonal anti-CD3 antibody, as a compelling justification for screening, as it can delay T1DM onset by 2–3 years. At the time this paper was written, teplizumab had been approved by the FDA only for use in the USA. Since then, the EMA has issued a positive opinion, and clinical availability of teplizumab is expected in the first or second quarter of 2026 in several European countries. However, approval has only been obtained for children > 8 years with stage 2 T1DM [42, 43]. Since it is not licensed for children younger than 8 years both in the USA and in Europe, the situation is unchanged for the majority of children targeted in pediatric screening initiatives (usually ages 2 and 6 years). Requiring 14 days of intravenous administration with intensive monitoring and premedication [44] presents major challenges since intravenous infusions, prolonged treatment duration, and premedication requirements can be physically and emotionally burdensome for children and families. Most patients experience mild to moderate adverse reactions that occur during or soon after the intravenous administration, with treatment discontinuation in 14.3% of cases due to adverse effects, such as lymphopenia, leukopenia, neutropenia, and cytokine-release syndrome (5.8%) [45, 46]. The response to this treatment is also highly variable; for example, children with ZnT8A are less likely to respond [42, 47]. While it appears safe in the long term, preserving C-peptide levels and improving metabolic parameters, its ultimate effects remain uncertain [48]. If proven safe and effective, the potential delay of T1DM onset could postpone the emotional and physical burdens on children and families and maintain their quality of life, potentially for a few more years [30]. While teplizumab is not generally available in the EU yet, an early diagnosis of T1DM (stage 1–2) allows for participation in research studies that could potentially delay clinical manifestation of T1DM [18].

### Monetary and personnel aspects

In the Fr1da study, screening costs ~ 28 Euros per child, with estimates decreasing to ~ 22 Euros when done population wide [49]. There is uncertainty on many costs, including laboratory expenses, which were negotiated for the Fr1da and ASK studies, but real-world prices may differ and are highly country and even region dependent. Medical follow-up, counseling, and education add significant costs that might not have been included in existing cost-analysis studies. Broken down, the screening itself would cost €33,400 for each child actually diagnosed with stage 3 T1DM or with a 50% risk of progressing to stage 3 within 2 years [23]. If eligible for teplizumab, an additional $193,900 (US price) would apply for the medication alone, with additional cost for injection of the drug during 14 days as well as medical surveillance [50]. Screening, follow-up, and potential teplizumab costs must be weighed against earlier insulin use and (DKA) hospitalization. A 20% decrease in DKA costs and 0.1% (1.1 mmol/mol) HbA1c improvement over a lifetime would be necessary to meet the $50,000–$150,000 per QALY threshold [51]. Therefore, cost-effective screening might only be possible in areas with high DKA prevalence and available screening capabilities. A US model showed the cost difference of 1,630,317 patients with T1DM compared to non-T1DM patients to be around $813 billion, meaning each patient with T1DM will cause medical expenses of around $500,000 throughout their lifetime. This argument will mainly come into play once there are potential cures for T1DM and not just a possible delay of a few years, such as with teplizumab (44).

The biggest issue regarding resource distribution is the staff required. Antibody screening needs clear communication and proper education for parents and children to ensure understanding and manage expectations [34]. Assuming pediatricians or family practitioners would conduct the screening, they will require both training and significant time for educating families, screening administration, and potentially lengthy follow-up appointments. Given the potential for high anxiety, adequate support must be available for all participants, likely requiring an expansion of existing medical resources [14].

## Discussion

General population screening for T1DM in children has rapidly gained traction with many studies discussing the medical aspects, but few dedicated to ethics. None of the publications identified in our study focused on the ethical aspects of this general population screening. To assess whether screening is warranted, the WHO recommends Wilson and Jungner’s framework [52, 53], which, while clinically focused, provides a good overview. Table 1 presents these criteria alongside Besser et al.’s [54] T1DM general population assessment and our ethical analysis based on Beauchamp and Childress’ approach. While there are other approaches as well, Beauchamp and Childress’ is internationally recognized and was developed for healthcare ethics issues, which is why it was used here. Besser et al.’s assessment was used because their modified criteria provide a more detailed framework than the standard criteria, e.g., those used by Achenbach et al. [14].

**Table 1:** Screening criteria from Wilson and Jungner, table adapted from Besser et al. (2022), and our ethical assessment

Modified Wilson and Jungner screening criteria	Assessment (by Besser et al.)	Ethical principle (and associated ethical concern, author assessment)
Condition should be an important health problem	Yes	
Target population for screening should be clearly defined and able to be reached	Uncertain	Justice: All families need to be aware of the possibility of screening and equally educated about the risks and benefits
Accepted treatment or course of action for patients who test positive that results in improved outcomes	Uncertain	Beneficence: Mainly limited to reduction of DKA rates; no available treatment (other than teplizumab) and no cure
Facilities for diagnosis and treatment should be available	No	Justice: No certainty whether all participants would have adequate access
Recognizable latent or early symptomatic stage	Yes	Nonmaleficence: Risk stratification not optimized yet
Suitable test or examination with appropriate performance characteristics	Uncertain	Justice: Optimized screening timepoints needed to maximize benefits and minimize healthcare expenditures
The test should be acceptable to the population	Uncertain	Nonmaleficence: Unclear data regarding anxiety levels and quality of life consequences for the child and parents
The screening test results should be clearly interpretable	Uncertain	Nonmaleficence: False sense of security if test is negative and unclear when/if T1DM will manifest if test is positive
Natural history of the condition should be adequately understood	Yes	Generally: Increase in T1D incidence and causality of the autoimmune process are not well understood
Cost of case finding (including diagnosis and treatment of patients diagnosed)	No	Justice: Cost-effectiveness is not clear especially regarding human resources; nationwide implementation and follow-up of high-risk patients would take resources from other important health problems
Overall benefit of the program should outweigh its harms	Uncertain	All: Ethically not justified at this time
Case finding should be a continuing process with ongoing monitoring and development	No	Justice: Ongoing monitoring needs to be available (human resources, medical resources, etc.)

### Autonomy

Autonomy is the ability to make personal, informed decisions, according to one’s own values and beliefs, without external interference [13]. Medical decisions made for children walk a fine line, as young children cannot make independent choices and are not legally allowed to, but depending on the age, children should be included in the decision-making process and asked for their opinion. The legal responsibility lies with the parent(s)/legal guardian(s), and while children often demonstrate health competency significantly earlier than the legal threshold [55], they will not exhibit this capacity if screening occurs at ages 2 and 6. Therefore, the legal guardian(s) will have to make the decision and need to be well educated about the topic which requires willingness to learn and access to resources tailored to their educational level. There is a widespread lack of knowledge about T1DM [56], and to remedy this, a major focus on education will be necessary, which must be accessible to the general population if T1DM antibody screening is to be extended. As children grow older, they should be more involved in their own health decisions and should be educated as well, but the screening and its consequences will have already taken place.

### Beneficence

Beneficence is the ethical principle of acting in the patient’s best interest, promoting their well-being and preventing harm [13]. One of the key advantages of general population T1DM screening is the early identification of children at risk of developing T1DM. Early identification is helpful in decreasing DKA risk, improving metabolic control, educating families and children about T1DM symptoms and treatment, and offering the opportunity of joining research studies [14, 18, 57]. So far, the only known effective treatment to delay stage 3 T1DM is teplizumab, which is not available in Europe yet and cannot be administered to children below 8 years (only off-label), who are at the greatest risk of DKA at clinical onset of T1DM [23]. Currently, there is no known or accepted treatment to actually prevent T1DM [14]. It is questionable whether the mentioned benefits warrant general population screening, especially since there are other approaches to reduce DKA at clinical onset, such as successful public awareness campaigns in Italy, Australia, Turkey, and Germany (Stuttgart Ketoacidosis Awareness Campaign) [54, 58, 59]. Another approach would be better education of physicians, as studies show that many children with diabetes-related symptoms have been seen by several physicians prior to the correct diagnosis. These other approaches should not be overlooked, as they could decrease DKA prevalence with potentially less ethical conflict.

### Nonmaleficence

Nonmaleficence is the ethical principle that requires healthcare providers to avoid harm to the patient [13]. Challenges of T1DM screening include the psychological impact associated with screening, uncertainty about optimal screening intervals, difficulty predicting disease onset, and lack of available treatment options. Parents of a child with a negative antibody screening (≤ 1 positive antibody) may be less inclined to seek medical advice, assuming T1DM is unlikely, which can lead to delayed diagnosis and increased DKA risk. Children of high-risk families might have more benefits from screening, due to higher disease and therefore DKA risk, but they might also have to be screened more often, with some studies recommending rescreening even annually, potentially increasing health anxiety. The psychological aspect is a major ethical concern. Although the Fr1da study reports no significant or lasting anxiety in parents of positively screened children, it is important to note that this conclusion is based on data derived from the PHQ-9 score, which primarily measures depression rather than anxiety. One study using the State Anxiety Inventory found elevated scores even 1 year after positive screening. Not only did they remain high, they were comparable with scores found in parents of children with clinical T1DM [32]. None of the studies looked at anxiety or stress in children participating in T1DM antibody screening programs. Additionally, lower parental education levels were associated with more anxiety, emphasizing the importance of educational material and communication targeting the family’s health literacy level. Psychological counseling should be available for all screening participants; however, the required intensity is unclear. Most importantly, screening knowledge and its consequences are irreversible, impacting the child’s life based on the legal guardian’s decision.

### Justice

Justice refers to fair, equitable, and appropriate distribution of benefits, risks, and costs within society [13]. To fulfill the ethical principle of justice, anyone must be allowed to participate. This requires the proper allocation of financial and HCP resources. The actual cost of general population screening has been estimated but does not account for teplizumab use, if available, and it is unclear whether the value threshold of $50,000–$150,000 per QALY gained would be met in all regions of the country. We did not identify any studies looking into the feasibility of this in Germany or other countries. However, a shortage of primary care pediatricians in Germany is evident and likely will get worse in the future. The same is true for the NHS in the UK. If a general screening program were to be initiated without expanding current resources, these resources would be lacking elsewhere, compromising treatment of other patients. Without ensuring equal and proper access to T1DM screening and without compromising other patient groups, the ethical principle of justice would not be met.

## Conclusion

Ethical implications of general population antibody screening for T1DM are multifaceted and require careful consideration. While the potential benefits of early detection and intervention can be significant, they must be weighed against the risks of psychological distress, stigmatization, and limited medical resources. Ongoing research and dialogue among healthcare providers, patients, and policymakers are essential to address the ethical challenges and ensure that screening practices align with the principles of autonomy, beneficence, nonmaleficence, and justice. This includes further research into the feasibility of general population T1DM screening regarding human resources, anxiety in parents and children, optimal screening timepoints, creation and verification of educational material targeting parents’ and children’s health literacy, appropriate degree of children’s involvement in T1DM screening, and development of additional options for delay or prevention of T1DM in the EU. Alternative options to reduce DKA incidence should also be investigated, as this is the primary benefit of screening at this time. Ultimately, a balanced approach that considers both medical and ethical dimensions will be crucial in the decision of whether to implement T1DM antibody screening in the general population. Right now, a general population screening outside of research studies is not ethically justified, and more clinical data is required to reassess. Since stakeholder perspectives were assessed solely through secondary sources though, we plan to address this limitation by conducting qualitative interviews with healthcare professionals, parents, and—where appropriate—the children themselves.

## Data Availability

No datasets were generated or analysed during the current study.
